# Conditional love? Co‐occurrence patterns of drought‐sensitive species in European grasslands are consistent with the stress‐gradient hypothesis

**DOI:** 10.1111/geb.13323

**Published:** 2021-05-31

**Authors:** Melinda M. J. de Jonge, Ana Benítez‐López, Stephan Hennekens, Luca Santini, Mark A. J. Huijbregts, Aafke M. Schipper

**Affiliations:** ^1^ Department of Environmental Science Institute for Water and Wetland Research Radboud University Nijmegen The Netherlands; ^2^ Integrative Ecology Group Estación Biológica de Doñana Consejo Superior de Investigaciones Científicas (EBD‐CSIC) Sevilla Spain; ^3^ Alterra – Vegetation, Forest and Landscape Ecology Alterra Wageningen UR Wageningen The Netherlands; ^4^ Institute of Research on Terrestrial Ecosystems National Research Council Monterotondo (Rome) Italy; ^5^ PBL – Netherlands Environmental Assessment Agency The Hague The Netherlands

**Keywords:** community ecology, drought stress, dry grasslands, joint species distribution model, species associations, stress‐gradient hypothesis

## Abstract

**Aim:**

The stress‐gradient hypothesis (SGH) postulates that species interactions shift from negative to positive with increasing abiotic stress. Interactions between species are increasingly being recognized as important drivers of species distributions, but it is still unclear whether stress‐induced changes in interactions affect continental‐to‐global scale species distributions. Here, we tested whether associations of vascular plant species in dry grasslands in Europe follow the SGH along a climatic water deficit (CWD) gradient across the continent.

**Location:**

Dry grasslands in Europe.

**Time period:**

Present.

**Major taxa studied:**

Vascular plants.

**Methods:**

We built a context‐dependent joint species distribution model (JSDM) to estimate the residual associations (i.e., associations that are not explained by the abiotic environment) of 161 plant species as a function of the CWD based on community data from 8,660 vegetation plots. We evaluated changes in residual associations between species for pairs and on the community level, and we compared responses for groups of species with different drought tolerances.

**Results:**

We found contrasting shifts in associations for drought‐sensitive and drought‐tolerant species. For drought‐sensitive species, 21% of the pairwise associations became more positive with increasing CWD, whereas 17% became more negative. In contrast, only 17% of the pairwise associations involving drought‐tolerant species became more positive, whereas 27% became more negative in areas with a high CWD. Additionally, the incidence of positive associations increased with drought for drought‐sensitive species and decreased for drought‐tolerant species.

**Main conclusions:**

We found that associations of drought‐sensitive plant species became more positive with drought, in line with the SGH. In contrast, associations of drought‐tolerant species became more negative. Additionally, changes in associations of single species pairs were highly variable. Our results indicate that stress‐modulated species associations might influence the distribution of species over large geographical extents, thus leading to unexpected responses under climate change through shifts in species associations.

## 1 INTRODUCTION

It has long been assumed that species distributions at large spatial scales are determined mainly by abiotic factors (Wisz et al., 2013). Nonetheless, there is increasing evidence that species interactions also influence large‐scale distributions of species (Staniczenko et al., 2018; Thuiller et al., 2015; Wisz et al., 2013). Given that these interactions might modulate the responses of species to environmental change, they could have important implications for predicting future species distributions (Wisz et al., 2013). However, disentangling the relative importance of species interactions and abiotic factors is challenging, particularly because abiotic conditions might alter the intensity and direction of the interactions (Bertness & Callaway, 1994; He et al., 2013). It is still unclear whether such environment‐dependent shifts in species interactions influence the continental‐to‐global scale distribution of species (Early & Keith, 2019; Wisz et al., 2013).

According to the stress‐gradient hypothesis (SGH), the frequency and importance of facilitative interactions in plant communities increases with increasing abiotic stress (Bertness & Callaway, 1994). This occurs through the amelioration of stressful conditions, for example when neighbouring individuals create shade that, in turn, reduces the evaporation of soil moisture in dry environments (Maestre et al., 2009). Many studies have provided evidence for the SGH along direct and indirect physical stress gradients, such as salinity and elevation (Bertness & Ewanchuck, 2002; Callaway et al., 2002), and for resource gradients, such as water availability (Dohn et al., 2013; He et al., 2013; Liancourt et al., 2005; López et al., 2016; Ziffer‐Berger et al., 2014), and biotic stress gradients, such as herbivory (Graff & Aguiar, 2011). Of these, the gradient in water availability is particularly relevant to investigate given the expected changes in the magnitude and frequency of droughts resulting from global climate change (Vicente‐Serrano et al., 2020).

Evidence for the SGH along water stress gradients is mixed, because several studies have found shifts from facilitative to competitive interactions under extreme drought stress (Berdugo et al., 2019; Maestre & Cortina, 2004; Soliveres et al., 2011; Soliveres & Maestre, 2014). This has led to refinements of the original formulation of the SGH, in which facilitative interactions are most prevalent in intermediately stressful conditions (Maestre et al., 2009; Michalet et al., 2014). Furthermore, few studies have investigated changes in interactions along large‐scale water stress gradients, and these have typically focused on community‐level metrics, such as biomass production (Dohn et al., 2013) and overall facilitation frequency or importance (Berdugo et al., 2019; Soliveres & Maestre, 2014), or cover only a few species (Ziffer‐Berger et al., 2014). However, community‐level responses to wide stress gradients can arise from the turnover of species with contrasting stress tolerances (Berdugo et al., 2019; Liancourt et al., 2017). Additionally, species with different life‐history strategies can show opposite responses to their neighbours, which might obscure community‐level patterns (Graff & Aguiar, 2017; Michalet et al., 2015). Hence, it remains unclear whether the large‐scale distributions of plant species are influenced by drought‐modulated species interactions and whether these follow expectations of the SGH.

Here, we tested whether shifts in plant species associations along the growing season climatic water deficit (CWD_GS_) gradient are consistent with the predictions of the SGH. We focused on dry grasslands in Europe, which encompass a wide water deficit gradient and are rich in species, hence including many possible species associations (Wilson et al., 2012). We fitted a context‐dependent joint species distribution model (JSDM; Tikhonov et al., 2017) to infer the pairwise associations of 161 species along the CWD_GS_. With this model, we identified residual associations between species, that is, those that cannot be explained directly by the abiotic environment, as a function of the CWD_GS_. We then investigated possible changes in associations along the CWD_GS_ for pairs of species and at the community level. We assessed associations for drought‐tolerant and drought‐sensitive species separately because a water deficit acts as a stress mainly for drought‐sensitive species, but not for drought‐tolerant species (Liancourt et al., 2005). We, therefore, hypothesized that with increasing water deficit, drought‐sensitive species would be increasingly facilitated by other plant species, resulting in more positive associations. In contrast, we expected that drought‐tolerant species would not be affected by the water deficit gradient, hence their associations would not change.

## 2 METHODS

### 2.1 Vegetation data

**FIGURE 1:**
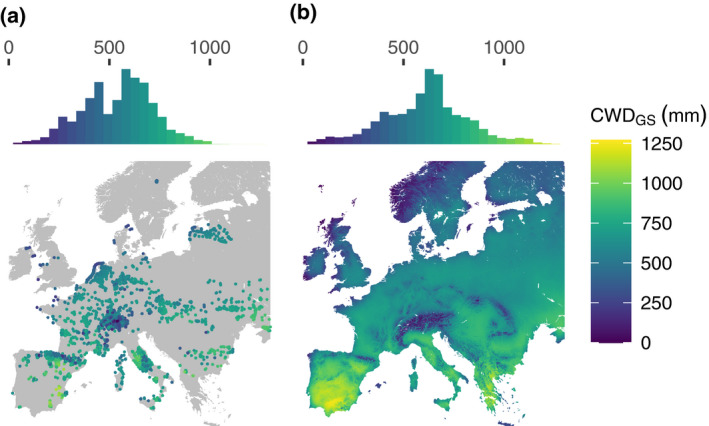
(a) Locations of the vegetation plots included in this study, coloured according to the climatic water deficit in the growing season (CWD_GS_; in millimetres) in the plot. (b) CWD_GS_ in Europe. The CWD_GS_ values are representative of 1979–2013. Histograms show the distribution of CWD_GS_ values over the plots (a) and in Europe (b)

We obtained occurrence data of herbaceous plant species in dry grasslands from the European Vegetation Archive (EVA; http://euroveg.org/eva‐database; Chytrý et al., 2016), which contains vegetation plot data from a large number of datasets in Europe. The vegetation plots, also called phytosociological relevés, include records of plant taxon co‐occurrence at particular sites at a high spatial resolution (generally between 4 and 25 m^2^), hence they can be considered suitable for analysis of co‐occurrence patterns in local communities. We selected plots classified as dry grasslands under the European Nature Information System (EUNIS) habitat classification. From these, we selected plots recorded between 1979 and 2013 and with a location uncertainty ≤ 1 km in order to match with the spatial and temporal resolution of the environmental variable data (Figure 1a; for a full overview of all databases included in this study, see Appendix 1; Supporting Information Table S1). This led to a dataset of 20,722 vegetation plots located in 8,660 unique 30 arc‐s grid cells (for the distribution of plots per grid cell, see Supporting Information Figure S1).

### 2.2 Environmental data

We selected 11 environmental variables that are known to influence plant distributions (Austin & Van Niel, 2011). To account for soil fertility, we included soil pH and soil cation exchange capacity (as centimoles of positive charge per kilogram of soil), which affect the potentially available nutrient supply (Chapin et al., 2011). Additionally, we included soil organic carbon content (per‐mille) as an indicator of soil texture and water infiltration rates and because it is an important nutrient reservoir (Chapin et al., 2011). We also included the following temperature variables (Prentice et al., 1992): maximum temperature of the warmest month (degrees Celsius), minimum temperature of the coldest month (degrees Celsius), growing degree days (GDD; > 5°C) and freezing degree days (FDD; < 0°C). Given that water availability for plants is not determined by total precipitation alone (Piedallu et al., 2013), we included the actual evapotranspiration (AET) in the growing season (AET_GS_; in millimetres) and the precipitation seasonality (coefficient of variation of monthly precipitation). We calculated monthly AET from monthly temperatures, monthly precipitation and available soil water capacity and summed monthly AET over the growing season (May–August) to obtain the AET_GS_ (Lutz et al., 2010; for details, see Supporting Information Appendix S1). Lastly, we included the climatic water deficit during the growing season (CWD_GS_; in millimetres) as an indicator of drought in our models (Figure 1b). We calculated the CWD_GS_ as the difference between the potential evapotranspiration during the growing season (PET_GS_, in milimeters) and the AET_GS_ (Lutz et al., 2010).

Soil variables are publicly available at a 30 arc‐s resolution (~1 km) at http://soilgrids.org (Hengl et al., 2014). Precipitation seasonality, temperature seasonality, maximum and minimum temperature and monthly temperature maps are also available at a 30 arc‐s resolution, as mean values from 1979 to 2013, from the CHELSA Climatologies dataset v.1.4 (http://chelsa‐climate.org; Karger et al., 2017). We calculated the GDD, FDD, PET_GS_, AET_GS_ and CWD_GS_ from mean monthly minimum, maximum and mean daily temperature and mean monthly precipitation averaged across 1978–2013 from the same dataset (for calculations, see Supporting Information Appendix S2).

### 2.3 Context‐dependent JSDM

We modelled species occurrence using a context‐dependent JSDM (Tikhonov et al., 2017). JSDMs are able to separate co‐occurrence patterns between species into shared environmental responses and residual associations that cannot be explained by the environment (Ovaskainen et al., 2017; Pollock et al., 2014). In original JSDMs, the estimated residual associations are static over space. However, recently, Tikhonov et al. (2017) developed a new context‐dependent latent variable approach, in which the residual associations are allowed to vary across the environment. We followed this approach and modelled species occurrences (*y*) as a probit regression:

(1) $$y_{\mathrm{ij}}=1_{z_{\mathrm{ij}}>0}$$

where *z_ij_* is the latent occurrence score for species *j* in vegetation plot *i*. This is calculated as (Tikhonov et al., 2017):

(2) $$z_{\mathrm{ij}}=\sum_{k=1}^{n_{c}}x_{\mathrm{ik}}\beta_{\mathrm{ij}}+\sum_{h=1}^{n_{h}}\eta_{\mathrm{ih}}\left(\lambda_{\mathrm{jh}}+\lambda_{\mathrm{jh}}^{\text{CWD}}{\text{CWD}}_{i}\right)$$

where *n_c_* is the number of environmental predictors plus intercept, β*_jk_* are the estimated regression coefficients, *x_ik_* are the measured environmental variables, *n_h_* is the number of latent variables, λ*_jh_* are the regression coefficients to the latent variables (latent loadings) and η*_ih_* is the latent factor site score. To calculate the residual associations between two species as a function of the CWD_GS_ (*R_j_*_1_ *_j_*_2,CWD_) we first calculated the covariance matrix of the species’ latent loadings as a function of the CWD_GS_ (Ω*_j_*_1_ *_j_*_2,CWD_) as:

(3) $$\Omega_{j_{1}j_{2},\text{CWD}}=\sum_{h=1}^{n_{h}}\left(\lambda_{j_{1}h}+\lambda_{j_{1}h}^{\text{CWD}}\text{CWD}\right)\left(\lambda_{j_{2}h}+\lambda_{j_{2}h}^{\text{CWD}}\text{CWD}\right)$$

We then transformed this covariance matrix into a correlation matrix to arrive at the residual associations (Equation 4).

(4) $$R_{j_{1}j_{2},\text{CWD}}=\frac{\Omega_{j_{1}j_{2},\text{CWD}}}{\sqrt{\Omega_{j_{1}j_{1},\text{CWD}}\Omega_{j_{2}j_{2},\text{CWD}}}}$$

We fitted the model using a Bayesian approach with an uninformative prior distribution, as described by Ovaskainen et al. (2017) and Tikhonov et al. (2017). We ran the model with three chains using a burn‐in period of 1,500,000 iterations, after which we sampled 1,250,000 iterations, of which we saved 5,000 iterations to construct the posterior of the model. We evaluated model convergence based on the maximum potential scale reduction factor and minimum effective sample size (Supporting Information Figures S3 and S4; Brooks & Gelman, 1998; Gelman & Rubin, 1992). A more detailed description of the model, the priors and the fitting procedure can be found in the Supporting Information (Appendix S3).

We accounted for collinearity between the environmental variables by excluding environmental variables with Pearson’s *r* > .7. In total, six of the initial environmental variables were retained: soil pH, soil cation exchange capacity, soil organic carbon content, precipitation seasonality, minimum temperature of the coldest month and CWD_GS_ (for correlations and selection rationale, see Supporting Information Figure S2). Given that plant species might show nonlinear responses to the environment, we also included the quadratic terms of each of the environmental variables. From each 30 arc‐s grid cell, we randomly selected only one plot to be included in the final model in order to prevent pseudo‐replication, leading to a total of 8,660 vegetation plots. Furthermore, we included only species with ≥ 10 presences per predictor in the selected vegetation plots (Peduzzi et al., 1996; leading to a minimum of 320 presences based on 12 environmental variables +20 latent variables). The final model included a total of 161 species, of which 35 were graminoids, 122 forbs and four shrubs (Supporting Information Table S2).

### 2.4 Testing the SGH

#### 2.4.1 Species groups

Given that we expected a difference in association shifts between drought‐tolerant and drought‐sensitive species, we divided the 161 species into three groups based on their Ellenberg values for soil moisture (E_m_). We classified species with E_m_ ≤ 3 as drought tolerant (DT; 36 species) and species with E_m_ ≥ 5 as drought sensitive (DS; 43 species) (terBraak & Gremmen, 1987). For all subsequent analyses, we split the results such that they included only pairwise associations where at least one of the species belonged to each of these two groups. We included also within‐group associations, because increasing abiotic stress can decrease competitive interactions when the competitive ability of stress‐sensitive dominant plant species is reduced (Liancourt et al., 2005). We considered species with 3 < E_m_ < 5 as intermediately tolerant and did not explicitly test the SGH for these species, because we did not have an a priori hypothesis regarding their responses. Note that the associations of intermediately tolerant species with drought‐tolerant or drought‐sensitive species were included in their respective analyses. Additionally, we tested a more stringent classification of species by considering only drought specialists (E_m_ ≤ 2.5; 13 species) and highly drought‐sensitive species (E_m_ ≥ 5.5; 25 species). We retrieved the Ellenberg values for soil moisture from a list compiled by Louette et al. (2010). To test whether the CWD_GS_ gradient is indeed a stress gradient for the drought‐sensitive but not for the drought‐tolerant species, we calculated the predicted number of species for both groups as a function of the CWD_GS_, with all other environmental variables set to their median value and without accounting for species associations. We calculated the number of species as the stacked probability of occurrence of all drought‐sensitive and drought‐tolerant species separately (Dubuis et al., 2011).

#### 2.4.2 Pairwise associations

**FIGURE 2:**
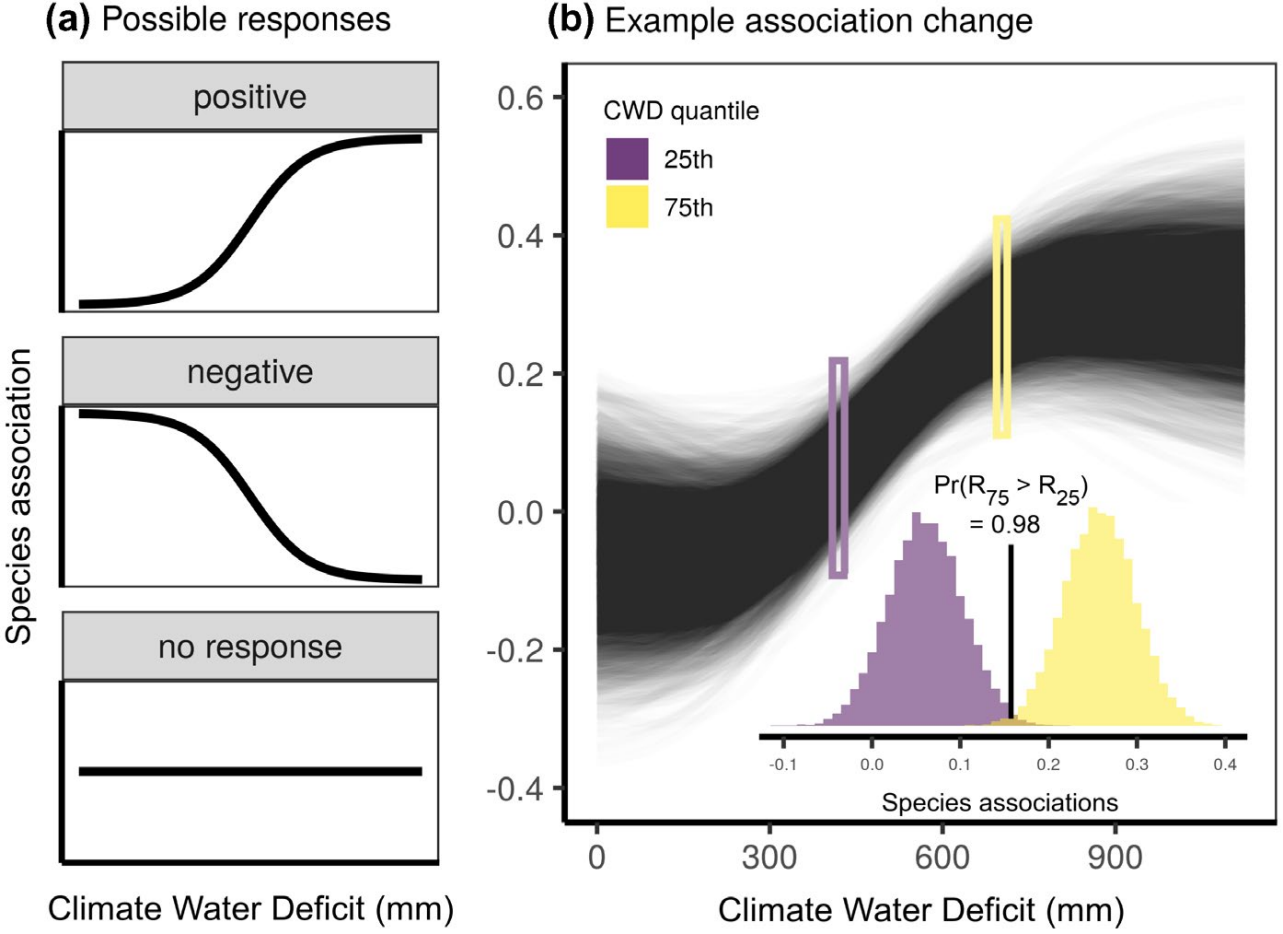
(a) Possible shapes of the relationship between pairwise species association and the growing season climatic water deficit (CWD_GS_) gradient considered in this study. (b) Example of how the response shape is determined for a given species pair, where black lines represent the full posterior of the pairwise association as a function of the CWD_GS_, and the purple and yellow histograms show the distribution of posterior values for the association at the 25th (420 mm) and 75th (665 mm) percentile of the CWD_GS_. Given that the Pr(*R*_75_ > *R*_25_) > .95, the association between these two species becomes significantly more positive with increased CWD_GS_, indicating increased facilitation with high water deficit

We first tested whether the association of each species pair shifted with the CWD_GS_. To this end, we checked, for each possible pair of species, whether their association changed along the full CWD gradient, thus excluding possible influences of species turnover on the results. We classified the relationship between the modelled residual association of a species pair and the CWD_GS_ into three main shifts (Figure 2a): positive, negative or no response. First, we extracted, for each species pair, the full posterior of the association at low CWD_GS_ (*R*_wet_; 420 mm, 25th percentile of the plots) and at high CWD_GS_ (*R*_dry_; 665 mm, 75th percentile of the plots). We then calculated the probability of the mean posterior value being significantly different at high CWD_GS_ compared with low CWD_GS_. We considered a shift in the association strength between species pairs positive for a probability (Pr) of *R*_dry_ > *R*_wet_ of > .95 and negative for a probability < .05 (Figure 1b). Associations for which .05 ≤ Pr(*R*_dry_ > *R*_wet_) ≤ .95 were considered to be indifferent to changes in the CWD_GS_. The choice to compare the associations at the 25th and 75th percentiles of the CWD_GS_ value can be considered conservative, because this corresponds to a relatively short stress gradient (He et al., 2013; but see also Soliveres & Maestre, 2014, who found that longer stress gradients are more conservative that shorter ones). To test the sensitivity of our results to this choice, we also performed the same analysis using the 5th (246 mm) and 95th (819 mm) percentiles of the CWD_GS_ gradient.

#### 2.4.3 Community associations

In addition to testing the shift in association strength for each pair of species, we analysed whether there is a higher overall proportion of positive compared with negative interactions with increasing CWD_GS_, summarized as the community association (CA):

(5) $${\text{CA}}_{\text{CWD}}=\frac{N_{\text{positive,CWD}}‐N_{\text{negative,CWD}}}{N_{\text{positive,CWD}}+N_{\text{negative,CWD}}}$$

where *N*_positive_ is the number of positive associations and *N*_negative_ is the number of negative associations at a given CWD. For *N*_positive_ and *N*_negative_, we included only associations for which the 95% credible interval did not overlap with zero. The value of CA_CWD_ ranges from minus one, where all significant associations are negative, to plus one, where all significant associations are positive. We did not use the association strengths retrieved from the models directly, because these have been shown to be biased by species prevalence and cannot be used to compare interaction strengths among species pairs (Zurell et al., 2018). We derived a separate CA for the drought‐sensitive and drought‐tolerant species groups such that, for each species within a group, significant associations with all the other species were included. Additionally, we calculated the CA for each possible combination of the two species groups (including only species combinations categorized as: DS + DS; DS + DT; DT + DT; DT + intermediate; and DS + intermediate).

For comparison, we also calculated the CA for the drought‐sensitive and drought‐tolerant species groups with a static version of the model, that is, one in which the regression coefficients of the species to the latent variables are not related to the CWD_GS_. We mapped the difference between this static model and the context‐dependent model (ΔCA = CA_static_ − CA) across Europe to identify areas where the context dependence of the associations is most prominent.

Lastly, we tested whether shifts in associations along the CWD_GS_ gradient were arising from shifts in the number of shared absences instead of shared presences. To this end, we calculated residuals from the unconditional predictions of the context‐dependent JSDMs and tested whether they were correlated with the CWD_GS_ (for more details, see Supporting Information Appendix S4). All model fitting and predictions were done in MATLAB v.R‐2017b using the “HSMC” package (HMSC 2.1 Matlab; Ovaskainen et al., 2017; Tikhonov et al., 2017). Analysis of model results, analysis of residuals and calculations of effective sample size was done in R v.3.4.4 (R Development Core Team, 2019). The code associated with this project can be found online at: https://github.com/MelindadeJonge/ConditionalLove

## 3 RESULTS

### 3.1 Pairwise associations

**FIGURE 3:**
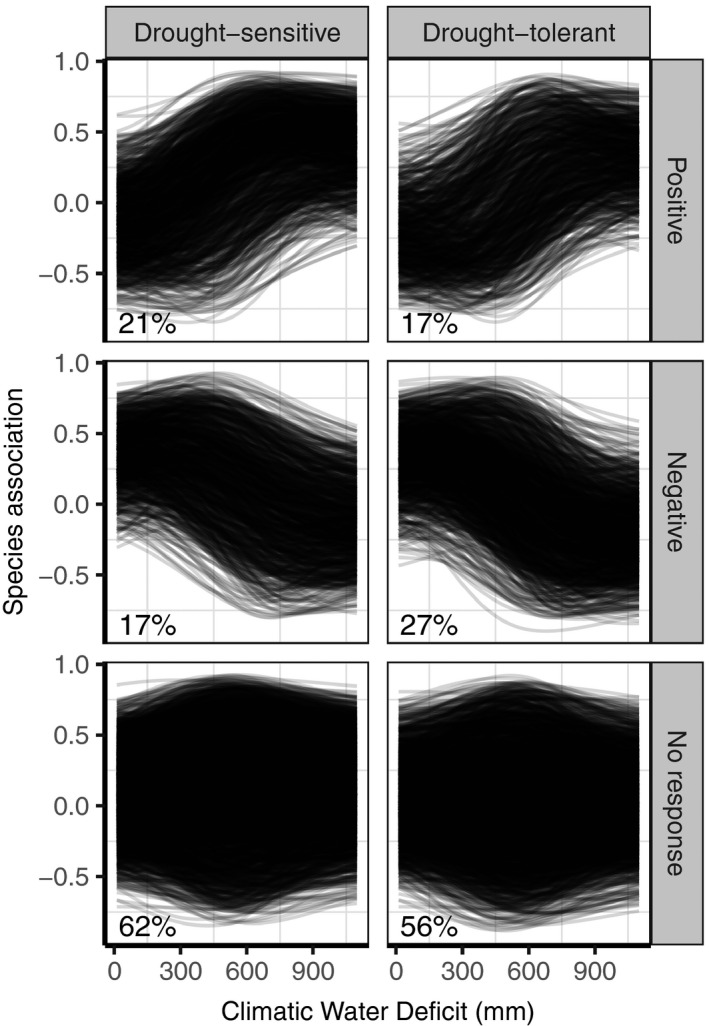
Mean residual association of each species pair involving one drought‐sensitive species (left panels) or one drought‐tolerant species (right panels) as a function of the growing season climatic water deficit (CWD_GS_) for three different response types (see Figure 2). Top panels show associations that are more positive at the 75th percentile (665 mm) of the CWD_GS_ than at the 25th percentile (420 mm). Middle panels show associations that become more negative with increasing CWD_GS_. Bottom panels show associations that do not change significantly along the CWD_GS_ gradient. Percentages indicate the percentage of associations per response type per species group. The CWD_GS_ gradient ranges from low (0 mm) to high water deficit (> 1,100 mm)

In accordance with our expectations, the pairwise associations of drought‐sensitive species more often increased (21%) than decreased (17%) with increasing CWD_GS_ (Figure 3, left panels). In contrast, the pairwise associations of drought‐tolerant species more often decreased   than increased (27% and 17% respectively; Figure 3, right panels). The majority of the pairwise associations (61% for drought‐sensitive and 56% for drought‐tolerant species) were indifferent to changes in CWD. When considering a wider CWD_GS_ gradient (5th–95th instead of 25th–75th percentiles), we found a slightly higher proportion of drought‐sensitive species with a positive response (23%), but otherwise the results were similar (Supporting Information Figure S5). A more stringent classification of species into highly drought sensitive and drought specialists yielded similar but more pronounced patterns with fewer negative shifts for highly drought‐sensitive species (15%) and fewer positive and more negative shifts for drought specialists (12% and 32%, respectively; Supporting Information Figure S6).

### 3.2 Community associations

**FIGURE 4:**
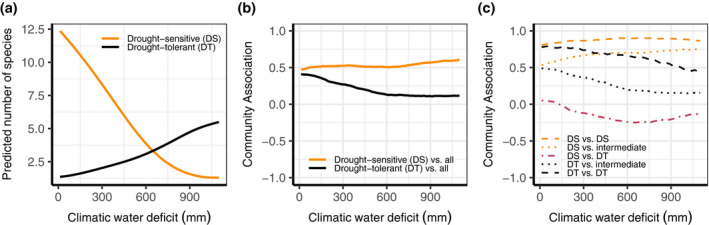
(a) The predicted number of drought‐sensitive species (DS; orange line) and drought‐tolerant species (DT; black line) as a function of the growing season climatic water deficit (CWD_GS_), with all other variables set at average values and excluding species associations. The number of species was calculated as the stacked probability of occurrence of all species belonging to that group. (b,c) Community association (CA) for (b) the two species groups overall and (c) between DS or DT species and species with intermediate tolerance (dotted), within groups (dashed) and between DT and DS species (dot‐dashed pink line). The CWD_GS_ gradient ranges from low (0 mm) to high water deficit (> 1,100 mm)

We found a reduction in the number of drought‐sensitive species and an increase in the number of drought‐tolerant species with increasing CWD_GS_ (Figure 4a). The CA for drought‐sensitive species increased with the CWD_GS_ in the second half of the gradient, that is, there were more positive compared with negative associations in dry than in wetter environments, which is in line with the expectations from the SGH (Figure 4b). This trend was mainly attributable to associations between drought‐sensitive and other species, rather than associations within the drought‐sensitive species group (Figure 4c). In contrast, the CA for drought‐tolerant species decreased with the CWD_GS_, whereas we expected the CA for this group to be indifferent to drought stress (Figure 4b). This trend reflected associations with species of intermediate sensitivity to drought in addition to associations within the species group itself (Figure 4c).

Associations between sensitive and tolerant species decreased with increasing drought along the first half of the gradient. Using the alternative Ellenberg indicator cut‐off values to classify the species into drought specialists and highly drought‐sensitive species revealed broadly similar patterns for sensitive species (Supporting Information Figure S7). The overall CA of drought specialists, in addition to the CA of drought specialists with intermediately tolerant species, followed a steeper decline over the gradient than those for drought‐tolerant species. Additionally, while associations within the drought‐tolerant group declined, associations within the drought specialist group did not change with the CWD_GS_ (Supporting Information Figure S7). In general, we found more positive than negative associations along the entire climatic water deficit gradient, that is, the CA was always positive, except for associations between drought‐sensitive and drought‐tolerant species (Figure 4b,c).

**FIGURE 5:**
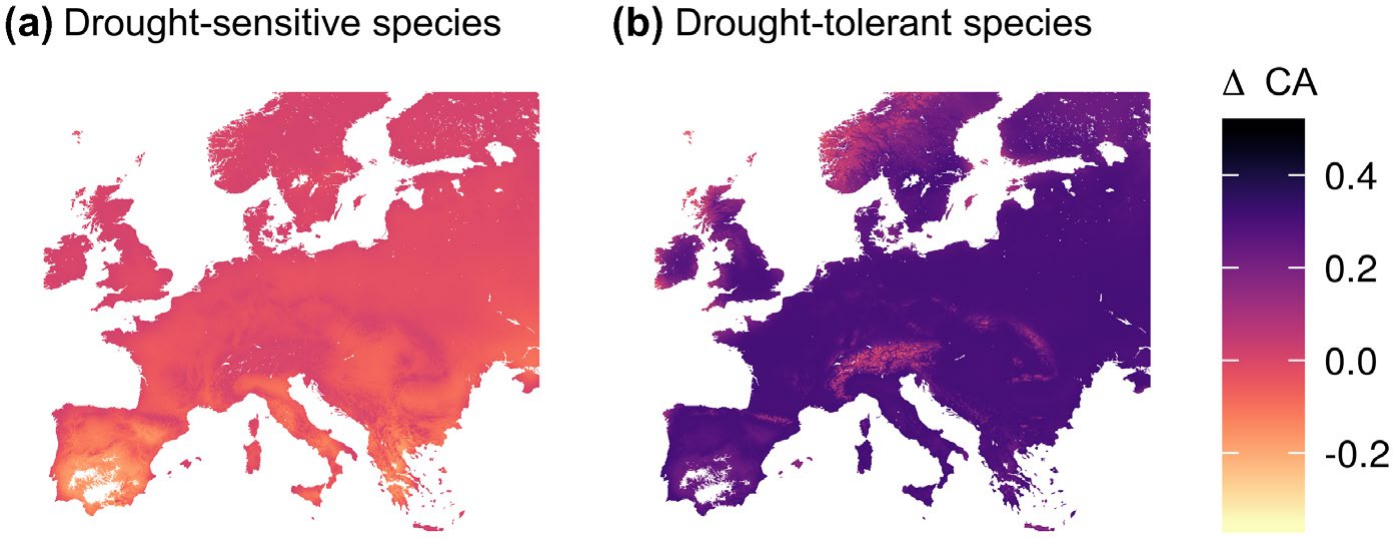
The difference in the overall community association (CA) between the CWD_GS_‐dependent joint species distribution model (JSDM) used in this study and a static JSDM (ΔCA = CA_static_ − CA) for associations of (a) drought‐sensitive species and (b) drought‐tolerant species in European dry grasslands. Positive values of ΔCA indicate an overestimation of associations in the static JSDM compared with the CWD_GS_‐dependent JSDM

Accounting for CWD_GS_ dependence changed the community associations for both species groups compared with a static JSDM (Figure 5). The static JSDM generally underestimated community associations involving drought‐sensitive species in dry regions, such as Spain and Greece (Figure 5a), indicating that the static model underestimates facilitation. Moreover, the static model overestimated the number of positive associations for drought‐tolerant species in the majority of the study area, except in relatively wet areas (e.g., in mountainous regions; Figure 5b). Lastly, we expect that the association shifts found are not induced by a shift in prevalence of shared absences, because the residuals of the unconditional model predictions were not correlated with the drought gradient (Supporting Information Figure S8).

## 4 DISCUSSION

Traditionally, species distributions have been considered to be determined mostly by abiotic factors, whereas our results suggest that biotic interactions also play a role and that these interactions might change along environmental gradients. More specifically, we showed that the species‐to‐species associations estimated from the distribution of plant species across European dry grasslands seem to be modulated by the CWD_GS_. We found contrasting patterns among groups of species with different drought tolerances. Drought‐sensitive species became more positively associated with intermediately drought‐tolerant species along the gradient, but not with drought‐tolerant species. In turn, drought‐tolerant species co‐occurred less with other species with increasing drought. Furthermore, individual species pairs often deviated from the SGH, because the majority of species‐to‐species associations did not shift along the gradient, indicating the importance of species‐specific factors other than stress tolerance. Our results conflict with the initial formulation of the SGH, which predicts a monotonic increase of positive interactions with increasing environmental severity (Bertness & Callaway, 1994). Instead, we found that changes in associations along the drought gradient depend on the stress tolerance of species, which is rather in line with refinements to the SHG (Graff & Aguiar, 2017; Maestre et al., 2009; Michalet et al., 2014).

We found a positive relationship between the predicted number of drought‐tolerant species and the CWD_GS_, indicating that increasing drought does not represent a stress gradient to these species. Furthermore, the proportion of positive associations for drought‐tolerant species, as indicated by the CA, decreased with increasing CWD_GS_. This might indicate an increased competition for water along the gradient, while these species do not benefit from facilitation. Similar results were found for stress‐tolerant shrubs and grasses in an experimental field study in an arid steppe (Graff & Aguiar, 2017). However, our results also indicate that drought‐tolerant species often do not act as nurses for drought‐sensitive species. For drought specialists (species with E_M_ < 2.5), the overall CA declined more strongly with CWD_GS_ compared with that of drought‐tolerant species (Figure 4b; Supporting Information Figure S7). Furthermore, the decline in the overall CA of drought‐tolerant species levelled off at intermediate CWD_GS_ values (600 mm), whereas the decline was steady over the whole gradient when only drought specialists were included. This might indicate that drought specialists are better competitors than other drought‐tolerant species in severely water limited environments. This agrees with recently found patterns along an aridity gradient in global drylands, where facilitation was most important in structuring communities with both tolerant and sensitive species, and competition was most important in structuring communities dominated by drought‐tolerant species (Berdugo et al., 2019). These patterns might lead to an increase in competition in severely water‐limited environments owing to species turnover from drought‐sensitive and intermediately sensitive species to drought‐tolerant species and drought specialists (Berdugo et al., 2019; Liancourt et al., 2017).

The CA of drought‐sensitive species exhibited little variation, with only a minor increase over the second half of the drought gradient (Figure 4b), meaning that the proportion of positive associations was similar at low and intermediate CWD_GS_ and increased thereafter. Associations between drought‐sensitive species and species of intermediate drought tolerance increased steadily along the gradient, whereas the associations between drought‐sensitive and drought‐tolerant species were similar at low and high CWD_GS_, with the lowest CA halfway along the gradient (Figure 4c). In contrast, associations between drought‐sensitive species themselves and between highly drought‐sensitive and drought‐specialist species did not vary along the gradient (Supporting Information Figure S7). These findings indicate that facilitation of drought‐sensitive species in dry environments is mainly governed by species with intermediate drought tolerance. Although neighbours might increase water availability in dry environments, they also compete for resources, and the outcome of an interaction is positive only when facilitative effects outweigh competition (Bimler et al., 2018; Michalet et al., 2014). Therefore, changes in the outcome of interactions along a gradient depend on how the competitive abilities and beneficial effects change along the gradient. Within this reasoning, our results might reflect that the competitive ability of drought‐tolerant species compared with other species increases more strongly along the CWD_GS_ than the benefits drought‐tolerant species might provide. Moreover, although the competitive abilities of species with intermediate drought tolerance compared with drought‐sensitive species might still increase along the CWD_GS_, the increased benefit of species with intermediate drought tolerance on drought‐sensitive species overcomes this, leading to more positive associations. Additionally, both drought‐sensitive species and drought specialists showed no change in their within‐group CA along the gradient. This is in line with earlier results showing that when fitness differences and niche overlap of two species remain similar over a gradient, their interaction does not vary either (Bimler et al., 2018). However, given that directionality cannot be estimated from co‐occurrence, nor does it provide estimates of fitness, these hypotheses cannot be tested with our approach.

Our results showed a large variation in association shifts among species pairs. The majority of species associations did not change significantly along the CWD_GS_ gradient. As such, it seems that associations of single species pairs do not necessarily comply with the SGH, although the frequency of positive associations within the community follows the SGH. This is in line with previous studies reporting that pairwise interactions are determined mainly by species‐specific traits (He et al., 2013) or life‐history stage (Losapio et al., 2019). This has also been proposed as an important factor underlying the conflicting evidence for the SGH in the past (He et al., 2013).

An interesting problem that might be explored in future studies is to determine which species traits, besides water stress tolerance, can predict association shifts. The traits that mediate the facilitation between species vary depending on the mechanism of facilitation. Under drought stress, plants might facilitate the growth of neighbouring plants by increasing soil moisture through hydraulic lift or providing shade, hence reducing evapotranspiration (Maestre et al., 2009). As such, nurse plants are expected to be shrubs, subshrubs and other large perennials with a large spread and deep root systems (Navarro‐Cano et al., 2021; Schöb et al., 2013). The two non‐sensitive shrub species included in this analysis, *Thymus praecox* and *Thymus pulegioides*, had positive shifts in their associations with more than half of the drought‐sensitive species (60% and 56%, respectively), which might indicate facilitation under drought by these species. Additionally, we found a high percentage of positive association shifts with drought‐sensitive species for *Salvia pratensis* (75%) and *Galium verum* (70%), which are both tall forbs of intermediate drought tolerance with a relatively wide aboveground spread. Furthermore, drought‐tolerant or intermediately tolerant graminoids rarely became more positively associated with sensitive species under drought stress. A notable exception was graminoid *Koeleria vallesiana*, which had positive association shifts with 55% of drought‐sensitive species. This species was recently identified as a potential nurse species in dry grasslands (Pescador et al., 2020), highlighting that there is a large variability in habits and traits of potential nurse plants.

Relating plant traits to interaction shifts is challenging, because shifts can arise not only from facilitation, but also from changes in the competitive ability of drought‐intolerant dominant species (Liancourt et al., 2005). For example, rather than acting as a nurse, the graminoid *Bromus erectus* (49% positive shifts with drought‐sensitive species) might have benefitted from reduced competition with increasing drought (Liancourt et al., 2005). Additionally, interactions also depend on the traits of the facilitated species and the similarity of traits of the interacting species, because nurses generally facilitate species with traits different from their own (He et al., 2013; Navarro‐Cano et al., 2021). Lastly, this picture is complicated further by the plasticity of these plant traits to plant fitness and life stage. In very stressful environments, the nurse plants might perform too poorly to realize the microclimatic habitat amelioration (Schöb et al., 2013). A systematic analysis of the prevalence of different traits along the drought stress gradient was beyond the scope of our analysis and might require locally measured trait data, given the plasticity of traits involved in the amelioration of drought stress. However, this would be an interesting avenue for further research. Additionally, given that the outcome of interactions depends on both competitive and facilitative effects, traits explaining differences in competitive abilities along the gradient, such as the Ellenberg indicator values of species to soil moisture used in the present study, also need to be included (Bimler et al., 2018).

Species associations in JSDMs indicate that two species co‐occur more or less frequently than expected from the abiotic environment. However, these associations are not necessarily indicative of species interactions. Residual associations can also arise from other sources, such as indirect interactions, common dispersal barriers and missing environmental covariates (Blanchet et al., 2020; D'Amen et al., 2018; Losapio et al., 2019; Ovaskainen et al., 2017; Tikhonov et al., 2017). We tried to overcome missing environmental covariates by including a broad set of environmental variables considered relevant for dry grassland plants. However, the resolution of these variables (30 arc‐s) is relatively coarse compared with the size of the vegetation plots and does not capture the local variability in topography and soil characteristics that affect soil moisture (Kemppinen et al., 2018). Unfortunately, we did not have high‐precision vegetation plot coordinates, and higher‐resolution environmental data would require locally acquired measurements per vegetation plot, which are not available. Furthermore, interactions between species generally act at the scale of individuals, and their impacts might vanish with increasing plot size (Thuiller et al., 2015), especially for competition (Rajaniemi et al., 2006). Although the vegetation plots used in the present study were designed to capture the local plant communities and were generally small in size, they might, nevertheless, be too coarse for certain interactions to be discernible. All in all, inferences on species interactions from species associations must be made with caution (Blanchet et al., 2020).

In the present study, we showed that the large‐scale distributions of plant species are mediated by shifts in the associations of species along a drought stress gradient. However, changes in species associations can also result from other stress gradients, such as nutrient availability, vapour pressure deficit and herbivory (Guignabert et al., 2020; He et al., 2013). Multiple stress gradients might lead to opposing patterns in species interactions, which can confound the effects of single gradients on species associations. For example, Guignabert et al. (2020) showed that seedlings of tree species can be facilitated by shrubs owing to the lower vapour pressure deficit under the canopy of the shrubs, even though soil moisture was reduced. This might increase facilitation in continental regions where the vapour pressure deficit is high whereas CWD_GS_ is low, such as the Alps. To understand fully how the distribution of species depends on species interactions, the effect of multiple stresses would need to be disentangled by modelling the associations as a function of all potentially important stress gradients. However, given that there are no clear expectations of how interacting stress gradients might alter species associations and because the availability of stress gradient data on this scale is limited, such an analysis is currently unfeasible.

Interactions between species are increasingly being recognized as important factors in shaping the distribution of species in addition to their response to varying environmental conditions on macroecological scales (Ockendon et al., 2014; Staniczenko et al., 2018; Wisz et al., 2013). Here we show, however, that accounting for these interactions is challenging, because species associations can shift along abiotic gradients. Consequently, changing environmental conditions might affect species not only directly, but also indirectly through changes in their interactions with other species. Facilitation in stressful environments can buffer the effect of increasing temperatures or drought on species distributions, which might lead current species distribution models to overestimate future climatically driven range losses of some species. For example, drought‐sensitive species might be facilitated by species with intermediate drought tolerance in mid‐ to high‐latitude regions in Europe, where moderate increases of drought are predicted (Vicente‐Serrano et al., 2020). Moreover, we found that drought specialists might outcompete drought‐tolerant species with increasing drought. This might, for example, lead to exacerbated losses of species if severe future drought is expected in areas with an already high CWD, such as in the Mediterranean region (Vicente‐Serrano et al., 2020). Lastly, given that nurse species might have expanded the distribution of species outside of their optimal environmental range (O’Brien et al., 2019), future loss of these species might have cascading effects on the distribution of facilitated species. Unfortunately, the use of co‐occurrence patterns does not allow us to determine the directionality of potential interactions, and the estimated associations between species cannot be used for predictions of future species distributions. We, therefore, conclude that stress‐modulated species interactions, such as those predicted by the SGH, might have unforeseen consequences for future species distributions in response to climate change.

## AUTHOR CONTRIBUTIONS

A.M.S., M.A.J.H. and M.M.J.d.J. developed the first ideas for this manuscript. A.M.S., L.S., M.A.J.H., A.B.‐L. and M.M.J.d.J. designed the research. S.H. provided and managed the vegetation data and collected Ellenberg values. M.M.J.d.J. performed the modelling work and data analysis. M.A.J.H., A.M.S., L.S., A.B.‐L. and M.M.J.d.J. interpreted and discussed the results. M.M.J.d.J. wrote the first draft of the manuscript, and all authors commented on various versions of the manuscript.

## BIOSKETCH

**Melinda de Jonge** is a PhD candidate at the Raboud University in The Netherlands. Her research focuses on disentangling the biotic and abiotic drivers of species distributions and modelling the impacts of anthropogenic activities on biodiversity. Her main interests are how interactions between species affect macroecological patterns of biodiversity and the responses of species to changes in the environment.

## Supporting information

Fig S1

Fig S2

Fig S3

Fig S4

Fig S5

Fig S6

Fig S7

Fig S8

Supplementary Material

## Data Availability

No new data were used in this study. All vegetation data were obtained from the European Vegetation Archives, and a list of sources and custodians of these data can be found in Appendix 1 and the Supporting Information. Climate and soil data were available from open and previously published sources.

## APPENDIX 1

### DATA SOURCES

Agrillo, E., Alessi, N., Massimi, M., Spada, F., De Sanctis, M., Francesconi, F., Cambria, V. E., & Attorre, F. (2017). Nationwide Vegetation Plot Database–Sapienza University of Rome: State of the art, basic figures and future perspectives. *Phytocoenologia*, ***47***, 221–229.

Apostolova, I., Sopotlieva, D., Pedashenko, H., Velev, N., & Vasilev, K. (2012). Bulgarian Vegetation Database: Historic background, current status and future prospects. *Biodiversity & Ecology*, ***4***, 141–148.

Biurrun, I., García‐Mijangos, I., Campos, J. A., Herrera, M., & Loidi, J. (2012). Vegetation‐plot database of the University of the Basque Country (BIOVEG). *Biodiversity & Ecology*, ***4***, 328.

Casella, L., Periangela, A. (2017). Vegetation database of Habitats in the Italian Alps – HabitAlp. *GIVD‐ID: EU‐IT‐010*.​ http://www.givd.info/ID/EU‐IT‐010

Chytrý, M., & Michalcová, D. (2012). Czech national phytosociological database. *Biodiversity & Ecology*, ***4***, 345–345.

Csiky, J., Botta‐Dukát, Z., Horváth, F., & Lájer, K. (2012). CoenoDat Hungarian Phytosociological Database. *Biodiversity & Ecology*, ***4***, 394–394.

Dengler, J., Rūsina, S., Boch, S., Bruun, H. H., Diekmann, M., Dierßen, K., Dolnik, C., Dupré, C., Golub, V. B., Grytnes, J., Helm, A., Ingerpuu, N., Löbel, S., Pärtel, M., Rašomavičius, V., Tyler, G., Znamenskiy, S., & Zobel, M. (2006). Working group on dry grasslands in the Nordic and Baltic region – Outline of the project and first results for the class Festuco‐Brometea. *Annali di Botanica N.S*., ***6***, 1–28.

Ewald, J., May, R., & Kleikamp, M. (2012). VegetWeb – The national online repository of vegetation plots from Germany. *Biodiversity & Ecology*, ***4***, 173–175.

Garbolino, E., De Ruffray, P., Brisse, H., & Grandjouan, G. (2012). The phytosociological database SOPHY as the basis of plant socio‐ecology and phytoclimatology in France. *Biodiversity & Ecology*, ***4***, 177–184.

Jandt, U., & Bruelheide, H. (2012). German vegetation reference database (GVRD). *Biodiversity & Ecology*, ***4***, 355–355.

Jansen, F., Dengler, J., & Berg, C. (2012). VegMV – The vegetation database of Mecklenburg‐Vorpommern. *Biodiveristy & Ecology*, ***4***, 149–160.

Kuzemko, A. (2012). Ukrainian grasslands database. *Biodiversity & Ecology*, ***4***, 430.

Onyshchenko V. A. (2009). *Forests of order Fagetalia sylvaticae in Ukraine*. Alterpress.

Rodwell, J. S. (2011). UK national vegetation classification database. *Biodiversity & Ecology*, ***4***, 381.

Rūsiņa, S. (2012). Semi‐natural grassland vegetation database of Latvia. *Biodiversity & Ecology*, ***4***, 409.

de Sanctis, M., Fanelli, G., Mullaj, A., & Attorre, F. (2017). Vegetation database of Albania. *Phytocoenologia*, ***46***, 107–108.

Schaminée, J. H., Hennekens, S. M., & Ozinga, W. A. (2012). The Dutch national vegetational database. *Biodiversity & Ecology*, ***4***, 201–209.

Šibík, J. (2012). Slovak vegetation database. *Biodiversity & Ecology*, *4*, 429.

Vassilev, K., Dajić, Z., Cušterevska, R., Bergmeier, E., & Apostolova, I. (2012). Balkan dry grasslands database. *Biodiversity & Ecology*, ***4***, 330.

Vassilev, K., Pedashenko, H., Alexandrova, A., Tashev, A., Ganeva, A., Gavrilova, A., Gradevska, A., Assenov, A., Vitkova, A., Grigorov, B., & Gussev, C. (2016). Balkan Vegetation Database: Historical background, current status and future perspectives. *Phytocoenologia*, ***46***, 89–95.

Vassilev, K., Ruprecht, E., Alexiu, V., Becker, T., Beldean, M., Csergő, A. M., & Öllerer, K. (2018). The Romanian Grassland Database (RGD): Historical background, current status and future perspectives. *Phytocoenologia*, ***48***, 91–100.

Venanzoni, R., Landucci, F., Panfili, E., & Gigante, D. (2012). Toward an Italian national vegetation database: VegItaly. *Biodiversity & Ecology*, ***4***, 185–190.

Willner, W., Berg, C., & Heiselmayer, P. (2012). Austrian vegetation database. *Biodiversity & Ecology*, ***4***, 333.
