# Supplementary figures and images for: Conditional love? Co‐occurrence patterns of drought‐sensitive species in European grasslands are consistent with the stress‐gradient hypothesis
Source: Glob Ecol Biogeogr. 2021 May 31;30(8):1609–20. doi: 10.1111/geb.13323 (PMC8362124; doi:10.1111/geb.13323)

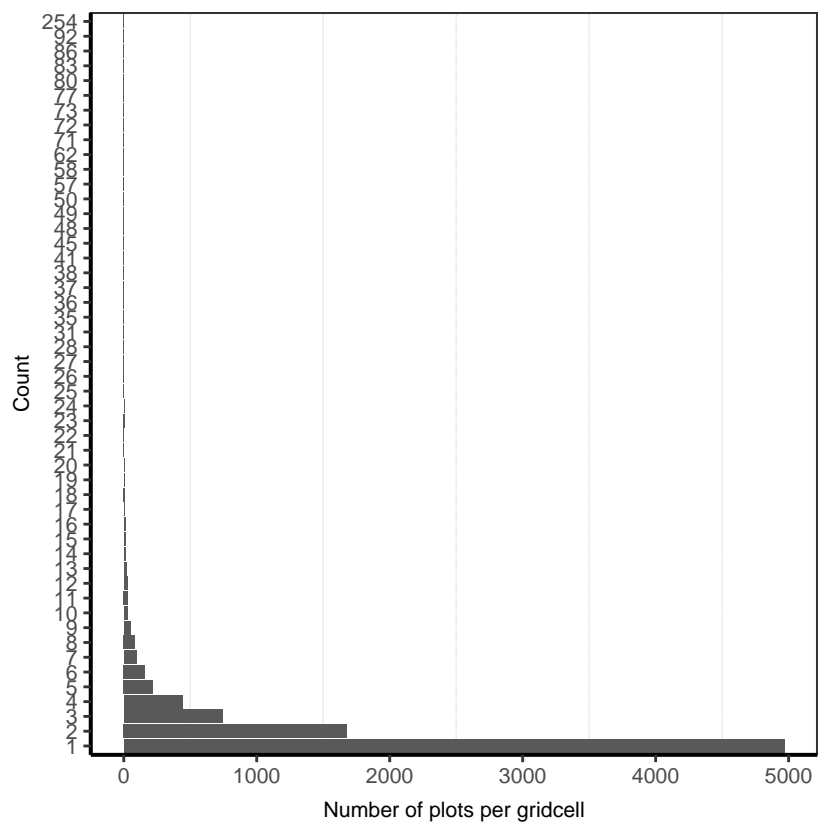

Supplement: Supplementary file 1 — Fig S1 [file GEB-30-1609-s004.pdf]

a)

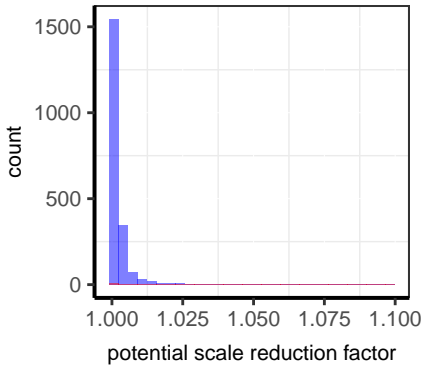

b)

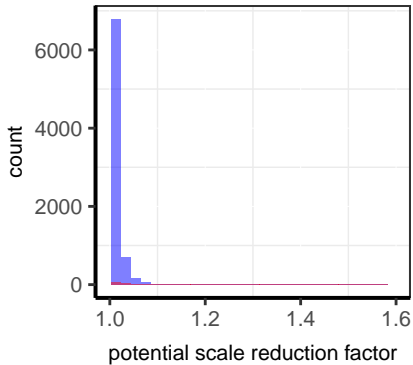

Supplement: Supplementary file 3 — Fig S3 [file GEB-30-1609-s003.pdf]

a)

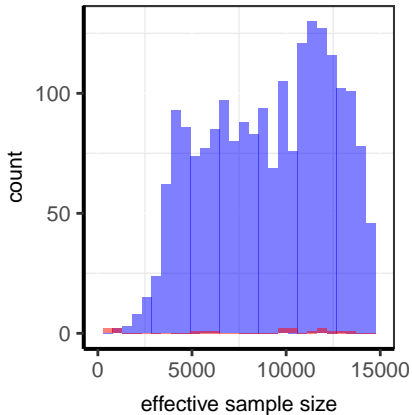

b)

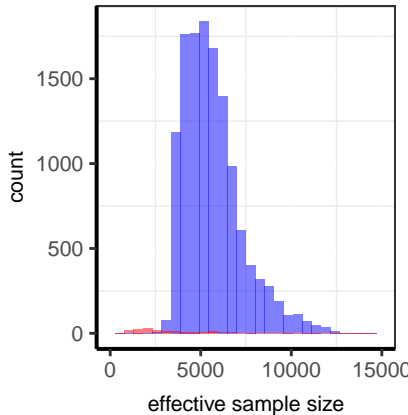

Supplement: Supplementary file 4 — Fig S4 [file GEB-30-1609-s009.pdf]

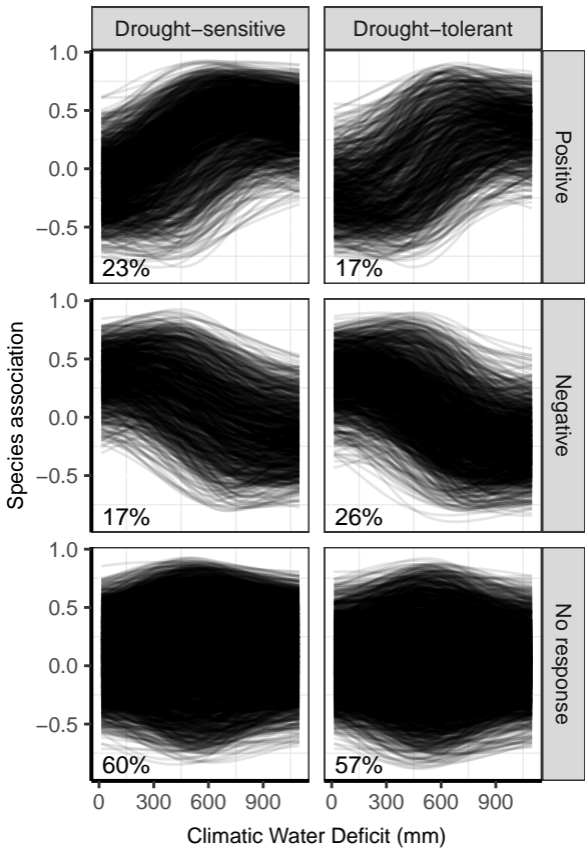

Supplement: Supplementary file 5 — Fig S5 [file GEB-30-1609-s007.pdf]

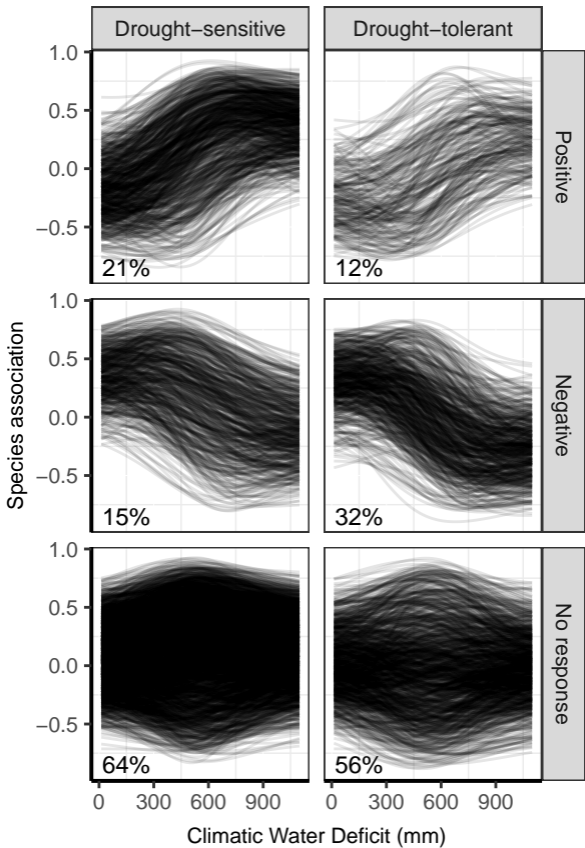

Supplement: Supplementary file 6 — Fig S6 [file GEB-30-1609-s008.pdf]

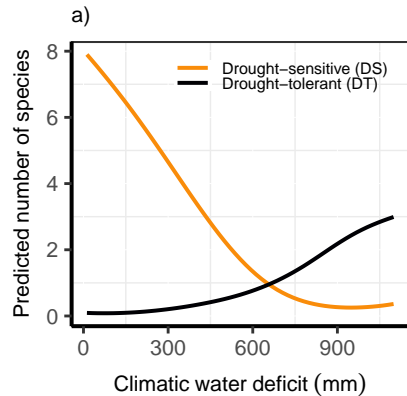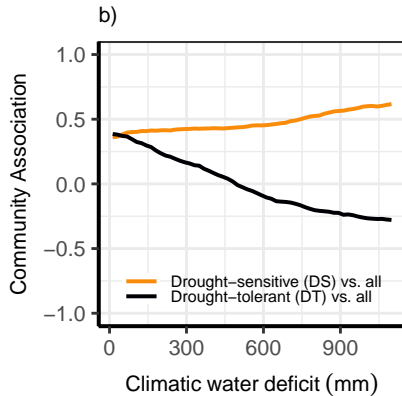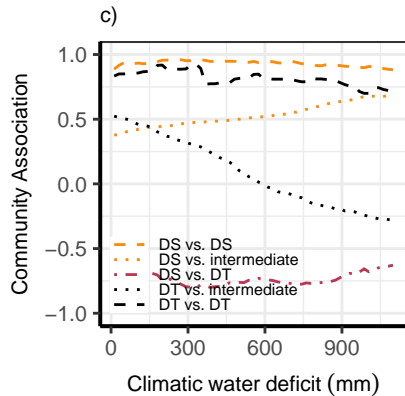

Supplement: Supplementary file 7 — Fig S7 [file GEB-30-1609-s005.pdf]

Briza media (slope:  $-4.2\text{e-}05$ )

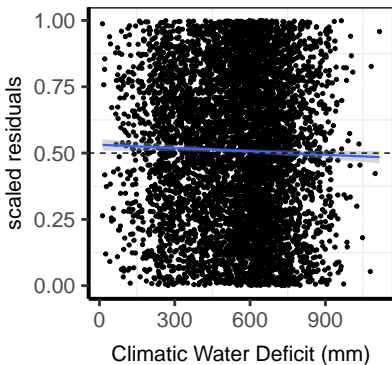

Carex humilis (slope:  $3.3\text{e-}05$ )

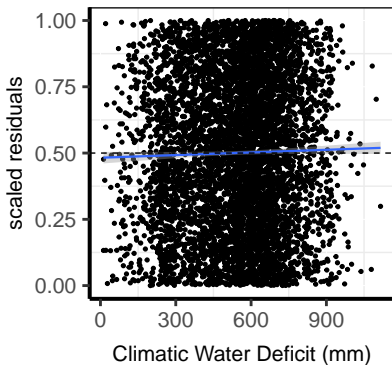

c)

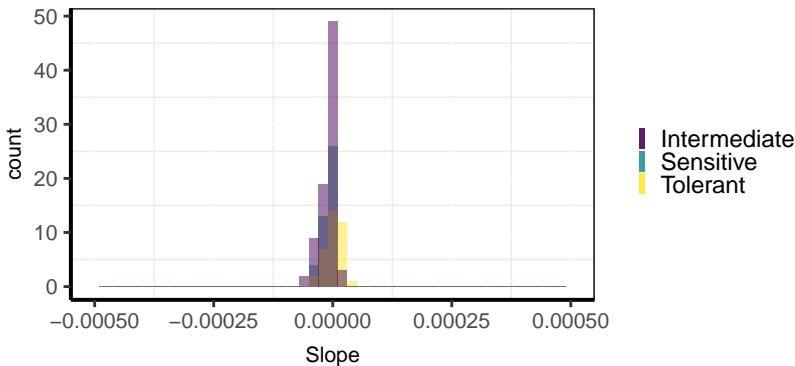

Supplement: Supplementary file 8 — Fig S8 [file GEB-30-1609-s006.pdf]
