# Supplementary material for: Conditional love? Co‐occurrence patterns of drought‐sensitive species in European grasslands are consistent with the stress‐gradient hypothesis
Source: Glob Ecol Biogeogr. 2021 May 31;30(8):1609–20. doi: 10.1111/geb.13323 (PMC8362124; doi:10.1111/geb.13323)
Supplement: Supplementary file 9 — Supplementary Material [file GEB-30-1609-s001.docx]

#### Supplementary material to:

***Conditional love? Co-occurrence patterns of drought-sensitive species in European grasslands are consistent with the stress-gradient hypothesis***

### Appendix S1: Potential and Actual EvapoTranspiration (PET & AET) and Climate Water Deficit (CWD)

Below, we give a step-by-step explanation of the water balance model used in this study to calculate the Actual Evapotranspiration, Soil Water Balance and Climate Water Deficit. This model is based on the work by Lutz, Van Wagtendonk and Franklin (2010). Input for the water balance model are monthly temperature maps from the CHELSA Climatologies dataset Version 1.4 (Karger et al., 2017) and the available soil water capacity of the top 100 cm soil layer available from http://soilgrids.org (Hengl et al., 2014).

###### Step 1: Potential evapotranspiration

We calculate the potential evapotranspiration (PET, mm/day) for each month using the Hargreaves method (Hargreaves & Allen, 2003).

${PET}_{m} = 0.0023{*RA}_{m}*(T_{mean,m}+17.8)*{{TD}_{m}}^{0.5}$ Eq. S1

Where TD_m_ is the difference between the maximum and the minimum temperature (in °C) and RA_m_ is the solar radiation (kJ/m^2^/day) on top of the atmosphere for the 15^th^ day of the month.

${RA}_{m} = \frac{24*60}{\pi}G_{sc}d_{r}(\omega_{s}sin(\varphi)sin(\delta)+cos(\varphi)sin(\omega_{s})$ Eq. S2

Where Gsc is the solar constant = 0.0820 MJ m^-2^ min^-1^, dr is the inverse relative Earth-Sun distance (Eq. S2.3), φ is the latitude in radians, δ is the solar decimation in radians and ω is the sunset hour angle in radians.

$d_{r} = 1+0.033cos\left( \frac{2\pi}{365}J \right)$ Eq. S3

$\delta= 0.409sin\left( \frac{2\pi}{365}J-1.39 \right)$ Eq. S4

$\omega=arccos(-tan(\varphi)tan(\delta))$ Eq. S5

###### Step 2: Monthly water balance

We used a simple water balance model to calculate the available water in each month using the equations from Lutz et al. (2010) as described here.

For each month, the precipitation in divided into a monthly rain (RAIN_m_, mm) and a monthly snow (SNOW_m_, mm):

${RAIN}_{m}=F_{m}*{Precipitation}_{m}$ Eq. S6

${SNOW}_{m} =(1-F_{m})*{Precipitation}_{m}$ Eq. S7

Where F_m_ is the montly melt factor which is equal to 0 for month with an average temperature (T_mean,m_) over 6 °C and 1 for months with an average temperature below 0 °C. For other months:

$F_{m}=0.167*T_{mean,m}$ for 0 °C <T_mean,m_< 6 °C Eq. S8

The melt factor F_m_ is also used to determine the monthly snowmelt (MELT_m_, mm):

${MELT}_{m} = F_{m}({SNOW}_{m}+{PACK}_{m-1})$ Eq. S9

Where PACK_m_ is the snow pack in a given month (in mm), calculated as:

${PACK}_{m}={(1-F_{m})}^{2}*{Precipitation}_{m}+(1-F_{m})*{PACK}_{m-1}$ Eq. S10

The total monthly water input (W_m_, mm) to the system is then:

$W_{m}={RAIN}_{m}+{MELT}_{m}$ Eq. S11

The available water in the soil (SOIL_m_, mm) is then calculated as:

${SOIL}_{m}=minimum\left\{ {SOIL}_{max},\left[ (W_{m}-{PET}_{m})+{SOIL}_{m-1} \right] \right\}$ Eq. S12

Where SOIL_max_ is the soil-water holding capacity of the top 100 cm of soil available from SoilGrids as the available soil water capacity (Hengl et al., 2014).

###### Step 3: Water availability variables

For each month the actual evapotranspiration (AET_m_, mm), climate water deficit (CWD_m_, mm) and soil water balance (SWB_m_, mm) were calculated according to equations Eq. S2.13 – Eq. S2.15.

${AET}_{m}={Precipitation}_{m}+({SOIL}_{m}-{SOIL}_{m-1})-{PET}_{m}$ Eq. S13

${CWD}_{m}={PET}_{m}-{AET}_{m}$ Eq. S14

${SWB}_{m}={Precipitation}_{m}+{SOIL}_{m}-{PET}_{m}$ Eq. S15

Growing season AET and CWD are calculated as the sum of monthly AET and CWD values between May and July.

### Appendix S2: Calculation of the growing and freezing degree days (GDD & FDD)

The growing degree days (GGD in °C) are calculated as sum of the average daily temperature of growing days between May and July. A growing day is defined as a day where the average temperature is above 5 °C.

$GDD = \sum_{d=121}^{212} \frac{T_{max,d}+T_{min,d}}{2}-T_{base}$ Eq. S16

With T_max,d_ is the maximum daily temperature (in °C), T_min,d_ is the minimum daily temperature(in °C)and T_base_ set equal to 5 °C. The freezing degree days are calculated similarly to the GDD except that we take the sum of the average daily temperature of freezing days over the whole year.

$GDD =-\sum_{d=1}^{365} \frac{T_{max,d}+T_{min,d}}{2}$ Eq. S17

Daily minimum and maximum temperatures are interpolated from monthly minimum and maximum temperatures averaged over 1979-2013 available from CHELSA ClimatologiesVersion 1.4 (Karger et al., 2017).

## Appendix S3: Model specifications

In this study we used the Joint Species Distribution Models developed by Tikhonov et al., (2017) and distributed under the HMSC 2.1 for MATLAB (https://www.helsinki.fi/en/researchgroups/statistical-ecology/software/old-versions-of-hmsc). Here, we present a summary of the model structure and the priors used in this study. For a full description including model considerations and MCMC sampling scheme we refer the reader to Tikhonov et al., (2017) and Ovaskainen et al., (2017)

#### CWD dependent model

Species occurrences (y) were modelled as a probit regression:

$y_{ij} = 1_{z_{ij}>0}$ Eq. S18

where z_ij_ are the latent occurrence scores for species j in vegetation plot i. These are calculated as (Tikhonov et al., 2017):

$z_{ij} = \sum_{k=1}^{n_{c}} x_{ik}\beta_{ij}+\varepsilon_{ij} + \epsilon_{ij}$ Eq. S19

where ncis the number of environmental predictors plus intercept, βjk are the estimated regression coefficients, xikare the measured environmental variable, ε_ij_represents the random effect which model the variation in species occurrences and species to species co-variation at the plot level (Eq. S21) and є_ij_ are the residuals modelled as є_ij_ ~ N(0,1).

The environmental regression coefficients β_ij_ follow a multivariate normal distribution (Ovaskainen and Soininen, 2011).

$\beta_{j\cdot} \sim N(\mu,V)$ Eq. S20

Here, β_j_ denotes the vector of regression coefficients for species j, μ represent the mean response of all species to the environmental covariates and V is a n_c_ x n_c_ variance-covariance matrix which measures how much the species vary in their responses on the diagonal and the off-diagonal elements give the covariances between responses to pairs of environmental covariates.

The random effect term is modelled as:

$\varepsilon_{ij} =\sum_{h=1}^{n_{h}} \eta_{ih}(\lambda_{jh}+\lambda_{jh}^{CWD}{CWD}_{i})$ Eq. S21

Where n_h_is the number of latent variables, λjh are the regression coefficients to the latent variables (latent loadings) and ηih are the latent factor site scores.

### Static model

The static JSDM follows the same structure as the context-dependent JSDM except for the random effect structure:

$\varepsilon_{ij} = \sum_{h=1}^{n_{h}} \eta_{ih}\lambda_{jh}$ Eq. S22

### Prior distributions

For both models, we used the default priors of the HMSC V2.1 MATLAB package (Tikhonov et al., 2017) that are shortly summarized here.

- For V we used an inverse-Wishart prior W^-1^(Ψ,v), where v=n_c_+1 and Ψ=I
- For each element of μ the prior is μ_k_ ~ N(0,1)
- The prior for the latent variables is η_ih_ ~ N(0,1)
- For the latent loadings $\lambda_{jh}$the package assumes a multiplicative gamma shrinkage prior following Bhattacharya & Dunson (2011)

$\lambda_{jh} \sim N\left( 0,\frac{1}{\tau_{h}\emptyset_{jh}} \right)$ Eq. S23

$\emptyset_{jh} \simГ\left( \frac{3}{2},\frac{3}{2} \right)$ Eq. S24

$\tau_{h} = \prod_{l=1}^{h} \delta_{l}$ Eq. S25

$\delta_{lk} \sim Ga(50,1)$ Eq. S26

- For $\lambda_{jh}^{CWD}$, the same priors were used as for $\lambda_{jh}$ (Eq. S23 – Eq. S26)

### Model convergence

We calculated the potential scale reduction factor (psrf, Gelman & Rubin, 1992, Brooks & Gelman, 1998) of the β and λ parameters in our model. Because, λ cannot be directly compared between chains we calculated the covariance matrix Ω at the 50^th^ percentile of the CWD, and evaluated the potential scale reduction factor of the covariance of all species pairs instead.

Additionally we calculated the effective sample size using the of the final model using the ‘mcmcse’ package in R (Flegal et al., 2017).

## Appendix S4: Analysis of residuals

In JSDMs, positive associations between species can arise from species being more often present together then expected based on the environmental variables alone as well as from species being absent together. Because of this, if a species is systematically over predicted by the environmental variables at high CWD, a found increase in the association with other species cannot be explained by increased facilitation along the gradient. We therefore tested whether the model residuals from the unconditional model predictions were biased along the CWD gradient using the following steps:

1. We calculated the scaled residuals using 3000 draws from the unconditional posterior predictions of the model using the ‘DHARMa’ package in R (Hartig, 2019). These scaled residuals range from 0 to 1 where 0 means that all predicted values are higher than the observed (overestimated, species is predicted to be present while it is absent), 0.5 means that half of the simulated values is higher and half is lower and a scaled residual of 1 means that all predicted values are lower than observed (underestimated, species is predicted to be absent while it is present).
2. We plotted the scaled residuals against the CWD for each gradient.
3. For each species, we calculated the slope of the scaled residuals against the CWD (scaled residuals ~ CWD).

## Figures


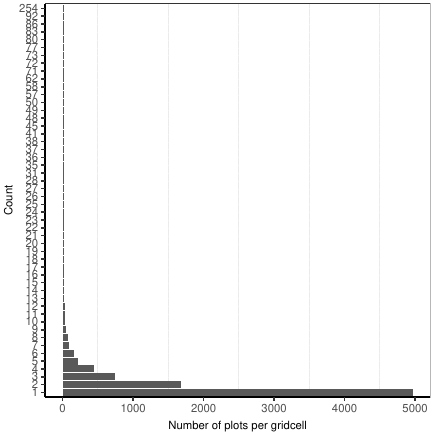


Figure S1: Histogram of the number of plots in each environmental grid cell. Only one plot per cell is used for the analysis.





Figure S2: Pearson correlations between the initial environmental variables included in this study. The Growing Degree Days (GDD), actual evapotranspiration (AET) and maximum temperature of the warmest month (Bio5) were excluded based on a high correlation with the CWD, i.e. the main variable of interest. Freezing Degree Days (FDD) was excluded from the final model due to a high correlation with the minimum temperature of the coldest month (Bio6).


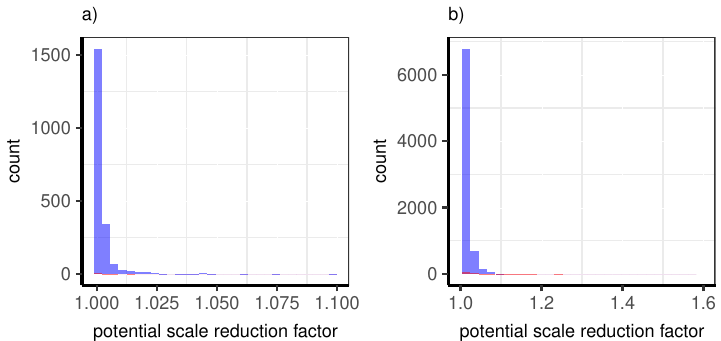


Figure S3: histograms of potential scale reduction factors (psrf) of context-dependent JSDM a) psrf of all beta parameters, b) psrf of omega estimates. Parameters of *Achillea millefolium* (in red) showed poor convergence, this species was excluded from our analyses.


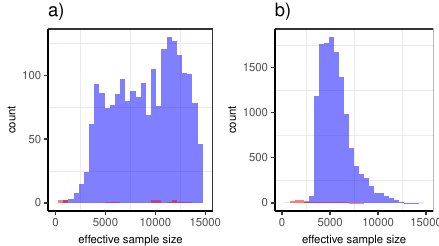


Figure S4: histograms of effective sample sizes (ESS) of context-dependent JSDM a) ESS of all beta parameters, b) ESS of omega estimates. Parameters of *Achillea millefolium* (in red) showed poor convergence, this species was excluded from our analyses.


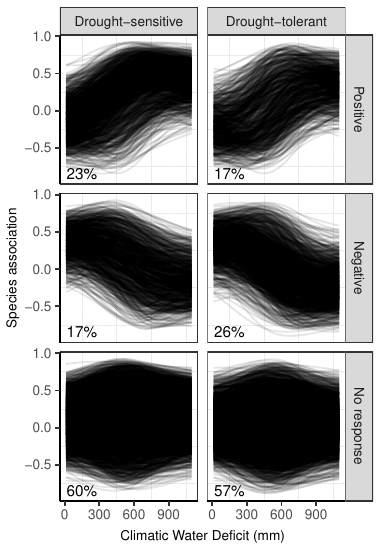


Figure S5: Mean residual association of each species pair involving one drought-sensitive species (DS, left) or one drought-tolerant species (DT, right) as a function of the growing season climatic water deficit (CWD_GS_) for three different response types (see Fig. 2). Upper panels show associations that are more positive at the 95th percentile (819 mm) of the CWD_GS_ than at the 5th percentile (246 mm), middle panels show the associations that become more negative with increasing CWD_GS_, and bottom panels show the associations that do not significantly change along the CWD_GS_ gradient. Percentages indicate the percentage of associations per response type per species group. The CWD_GS_ gradient ranges from low (0 mm) to high water deficit (>1100 mm). Drought-sensitive species are species with an Ellenberg `indicator value for moisture ≥ 5 while drought-tolerant species have an Ellenberg indicator value for moisture ≤ 3.


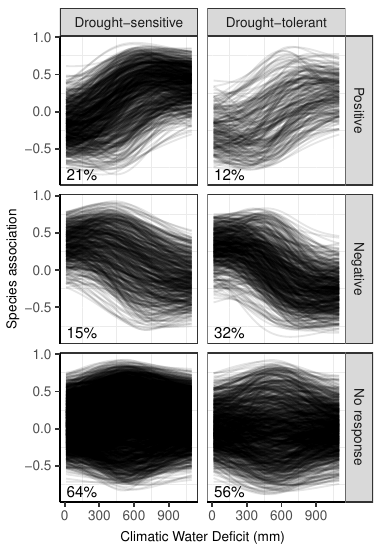


Figure S6: Mean residual association of each species pair involving one drought-sensitive species (DS, left) or one drought-tolerant species (DT, right) as a function of the growing season climatic water deficit (CWD_GS_) for three different response types (see Fig. 2). Upper panels show associations that are more positive at the 75th percentile (665 mm) of the CWD_GS_ than at the 25th percentile (420 mm), middle panels show the associations that become more negative with increasing CWD_GS_, and bottom panels show the associations that do not significantly change along the CWD_GS_ gradient. Percentages indicate the percentage of associations per response type per species group. The CWD_GS_ gradient ranges from low (0 mm) to high water deficit (>1100 mm). Drought-sensitive species are species with an Ellenberg indicator value for moisture ≥ 5.5 (25 species) while drought-tolerant species have an Ellenberg indicator value for moisture ≤ 2.5 (13 species).


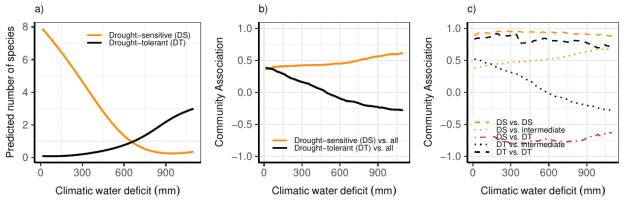


**Figure S7: a) The predicted number of drought-sensitive species (orange line) and drought-tolerant species (black line) as a function of the growing season climatic water deficit (CWDGS), with all other variables set at average values and excluding species’ associations. The number of species was calculated as the stacked probability of occurrence of all species belonging to that group. b) Community association (CA) for the two species groups overall and c) between DS or DT species and intermediately tolerant species (dotted), within groups (dashed) and between DT and DS species (dot-dashed pink line). The CWDGS gradient ranges from low (0 mm) to high water deficit (>1100 mm). Drought-sensitive species are species with an Ellenberg indicator value for moisture ≥ 5.5 (25 species) while drought specialist species have an Ellenberg indicator value for moisture ≤ 2.5 (13 species).**


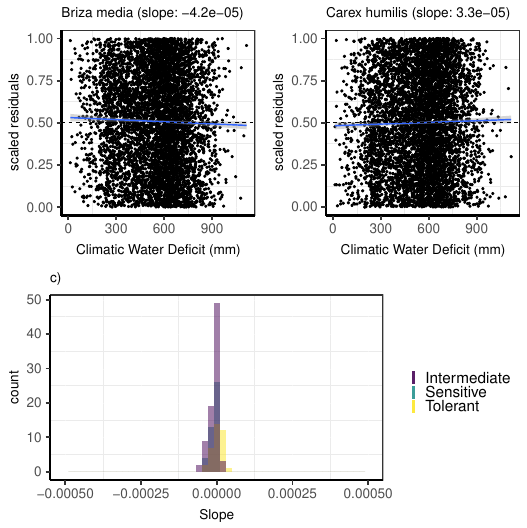


Figure S8: a & b) Examples of the distribution of scaled residuals along the climatic water deficit (CWD) for 4 species in our model, c) histogram of found linear regression slopes of scaled residuals of the fixed effects part of the model with the CWD for all species (n = 161, mean = -0.00002, min = *-0.00006, max = 0.00002)*). Scaled residuals were calculated from the unconditional model predictions (predictions based on only environmental variables) using the DHARMa package in R.

## Tables

Table S1: Overview of the datasets included in this study.

| Dataset | Custodian | Dep. custodian | Plots |
| --- | --- | --- | --- |
| Albanian Vegetation Database | Michele de Sanctis | Giuliano Fanelli | 30 |
| Austrian Vegetation Database | Wolfgang Willner | - | 781 |
| Balkan Dry Grassland Database | Kiril Vassilev | Armin Macanović | 282 |
| Balkan Vegetation Database | Kiril Vassilev | Hristo Pedashenko | 27 |
| Basque Country Database | Idoia Biurrun | Itziar Garcia-Mijangos | 1059 |
| UK National Vegetation Classification Database | John S. Rodwell | - | 104 |
| Bulgarian Vegetation Database | Iva Apostolova | Desislava Sopotlieva | 1 |
| Czech National Phytosociological Database | Milan Chytry | Dana Michalcova | 242 |
| SOPHY | Henry Brisse | Patrice de Ruffray | 717 |
| German Vegetation Reference Database (GVRD) | Ute Jandt | Helge Bruelheide | 1468 |
| VegetWeb Germany | Florian Jansen | Jörg Ewald | 605 |
| VegMV | Florian Jansen | Christian Berg | 74 |
| CoenoDat Hungarian Phytosociological Database | János Csiky | Zoltán Botta-Dukát | 211 |
| Vegetation database of Habitats in the Italian Alps - HabItAlp | Laura Casella | Pierangela Angelini | 21 |
| Vegetation Plot Database - Sapienza University of Rome | Emiliano Agrillo | Fabio Attorre | 1069 |
| Semi-natural Grassland Vegetation Database of Latvia | Solvita Rusina | - | 901 |
| Dutch National Vegetation Database | Joop Schaminée | Stephan Hennekens | 3183 |
| Nordic-Baltic Grassland Vegetation Database (NBGVD) | Jurgen Dengler | Łukasz Kozub | 341 |
| Romania Grassland Database | Eszter Ruprecht | Kiril Vassilev | 53 |
| Slovak Vegetation Database | Milan Valachovič | Jozef Šibík | 123 |
| Iberian and Macaronesian Vegetation Information System (SIVIM) - Alpine | Borja Jiménez-Alfaro | Xavier Font Castell | 155 |
| Iberian and Macaronesian Vegetation Information System (SIVIM) - Grasslands | Maria Pilar Rodriquez-Rojo | Xavier Font Castell | 590 |
| Iberian and Macaronesian Vegetation Information System (SIVIM) - Scrubs | Rosario G. Gavilán | Xavier Font Castell | 1 |
| Iberian and Macaronesian Vegetation Information System (SIVIM) - Wetlands | Aaron Pérez-Haase | Xavier Font Castell | 12 |
| Switzerland grasslands | - | - | 7679 |
| Ukraine Grassland Database | Anna Kuzemko | Yulia Vashenyak | 863 |
| Vegetation Database of Ukraine and Adjacent Parts of Russia | Viktor Onyshchenko | Vitaliy Kolomiychuk | 107 |
| VegItaly | Roberto Venanzoni | Flavia Landucci | 23 |

Table S2: Overview of the species included in the model and their Ellenberg value for moisture. Species followed by an asterisk showed poor parameter convergence and were removed from analysis of model results.

| ***Species*** | ***Family*** | ***Habit*** | ***Ellenberg M*** |
| --- | --- | --- | --- |
| *Achillea millefolium** | Compositae | Forb | 4.2 |
| *Agrimonia eupatoria* | Rosaceae | Forb | 4 |
| *Agrostis capillaris* | Poaceae | Graminoid | 4.5 |
| *Aira praecox* | Poaceae | Graminoid | 2.3 |
| *Ajuga reptans* | Lamiaceae | Forb | 6.2 |
| *Alchemilla vulgaris agg.* | Rosaceae | Forb | 5.7 |
| *Antennaria dioica* | Compositae | Forb | 4.2 |
| *Anthericum liliago* | Asparagaceae | Forb | 3 |
| *Anthoxanthum odoratum* | Poaceae | Graminoid | 6 |
| *Aquilegia atrata/vulgaris* | Ranunculaceae | Forb | 4 |
| *Arabis hirsuta* | Brassicaceae | Forb | 4.1 |
| *Arenaria serpyllifolia* | Caryophyllaceae | Forb | 3.8 |
| *Arrhenatherum elatius* | Poaceae | Graminoid | 5 |
| *Artemisia campestris* | Compositae | Forb | 2.5 |
| *Asperula cynanchica* | Rubiaceae | Forb | 3 |
| *Astrantia major* | Apiaceae | Forb | 6 |
| *Avenula pubescens* | Poaceae | Graminoid | 4 |
| *Bellis perennis* | Compositae | Forb | 5 |
| *Brachypodium pinnatum* | Poaceae | Graminoid | 3.7 |
| *Briza media* | Poaceae | Graminoid | 5 |
| *Bromopsis erecta* | Poaceae | Graminoid | 3.2 |
| *Buphthalmum salicifolium* | Compositae | Forb | 4 |
| *Calamagrostis epigejos* | Poaceae | Graminoid | 7 |
| *Calluna vulgaris* | Ericaceae | Shrub | 6 |
| *Campanula glomerata* | Campanulaceae | Forb | 4 |
| *Campanula rotundifolia* | Campanulaceae | Forb | 4.3 |
| *Carduus defloratus* | Compositae | Forb | 4 |
| *Carex arenaria* | Cyperaceae | Graminoid | 3.2 |
| *Carex caryophyllea* | Cyperaceae | Graminoid | 4 |
| *Carex flacca* | Cyperaceae | Graminoid | 5.9 |
| *Carex humilis* | Cyperaceae | Graminoid | 2.6 |
| *Carex montana* | Cyperaceae | Graminoid | 4.4 |
| *Carex sempervirens* | Cyperaceae | Graminoid | 4 |
| *Carlina acaulis* | Compositae | Forb | 4 |
| *Carlina vulgaris* | Compositae | Forb | 4.6 |
| *Carum carvi* | Apiaceae | Forb | 5 |
| *Centaurea jacea* | Compositae | Forb | 4 |
| *Centaurea scabiosa* | Compositae | Forb | 3 |
| *Cerastium arvense* | Caryophyllaceae | Forb | 4 |
| *Cerastium fontanum* | Caryophyllaceae | Forb | 5 |
| *Cerastium semidecandrum* | Caryophyllaceae | Forb | 3.3 |
| *Cirsium acaulon* | Compositae | Forb | 3.2 |
| *Clinopodium acinos* | Lamiaceae | Forb | 2 |
| *Clinopodium alpinum* | Lamiaceae | Forb | 5 |
| *Clinopodium vulgare* | Lamiaceae | Forb | 3.9 |
| *Colchicum autumnale* | Colchicaceae | Forb | 6 |
| *Convolvulus arvensis* | Convolvulaceae | Forb | 4 |
| *Corynephorus canescens* | Poaceae | Graminoid | 2.2 |
| *Cynosurus cristatus* | Poaceae | Graminoid | 5 |
| *Dactylis glomerata* | Poaceae | Graminoid | 4.9 |
| *Dactylorhiza maculata* | Orchidaceae | Forb | 7.2 |
| *Danthonia decumbens* | Poaceae | Graminoid | 6 |
| *Daucus carota* | Apiaceae | Forb | 4 |
| *Dianthus carthusianorum* | Caryophyllaceae | Forb | 3.2 |
| *Dianthus sylvestris* | Caryophyllaceae | Forb | 3.5 |
| *Echium vulgare* | Boraginaceae | Forb | 3.8 |
| *Erodium cicutarium* | Gereniaceae | Forb | 3.7 |
| *Erophila verna* | Brassicaceae | Forb | 2.7 |
| *Eryngium campestre* | Apiaceae | Forb | 3 |
| *Euphorbia cyparissias* | Euphorbiaceae | Forb | 3 |
| *Euphorbia verrucosa* | Euphorbiaceae | Forb | 3 |
| *Festuca filiformis* | Poaceae | Graminoid | 3.8 |
| *Festuca valesiaca* | Poaceae | Graminoid | 2.3 |
| *Filipendula vulgaris* | Rosaceae | Forb | 3.5 |
| *Fragaria vesca* | Rosaceae | Forb | 4.8 |
| *Fragaria viridis* | Rosaceae | Forb | 3.3 |
| *Galium lucidum* | Rubiaceae | Forb | 3 |
| *Galium mollugo* | Rubiaceae | Forb | 4.2 |
| *Galium pumilum* | Rubiaceae | Forb | 4 |
| *Galium verum* | Rubiaceae | Forb | 4 |
| *Gentiana lutea* | Gentianaceae | Forb | 4.8 |
| *Gentiana verna* | Gentianaceae | Forb | 4 |
| *Geranium sylvaticum* | Geraniaceae | Forb | 5.6 |
| *Globularia nudicaulis* | Plantaginaceae | Forb | 4 |
| *Gymnadenia conopsea* | Orchidaceae | Forb | 6.3 |
| *Helianthemum nummularium* | Cistaceae | Forb | 3.3 |
| *Helianthemum oelandicum* | Cistaceae | Forb | 3 |
| *Heracleum sphondylium* | Apiaceae | Forb | 5 |
| *Hieracium murorum* | Compositae | Forb | 5 |
| *Hippocrepis comosa* | Fabaceae | Forb | 2.8 |
| *Holcus lanatus* | Poaceae | Graminoid | 6 |
| *Hypericum perforatum* | Hypericaceae | Forb | 4 |
| *Hypochaeris radicata* | Compositae | Forb | 4.3 |
| *Jacobaea vulgaris* | Compositae | Forb | 4 |
| *Jasione montana* | Campanulaceae | Forb | 3.2 |
| *Knautia arvensis* | Caprifoliaceae | Forb | 3.8 |
| *Koeleria macrantha* | Poaceae | Graminoid | 3.8 |
| *Koeleria pyramidata* | Poaceae | Graminoid | 4.3 |
| *Koeleria vallesiana* | Poaceae | Graminoid | 1 |
| *Laserpitium latifolium* | Apiaceae | Forb | 5 |
| *Lathyrus pratensis* | Fabaceae | Forb | 6 |
| *Leontodon hispidus* | Compositae | Forb | 4.5 |
| *Leucanthemum vulgare* | Compositae | Forb | 4 |
| *Linum catharticum* | Linaceae | Forb | 5 |
| *Lotus corniculatus* | Fabaceae | Forb | 4 |
| *Luzula campestris* | Juncaceae | Graminoid | 4 |
| *Medicago falcata* | Fabaceae | Forb | 3 |
| *Medicago lupulina* | Fabaceae | Forb | 4 |
| *Molinia caerulea* | Poaceae | Graminoid | 7.3 |
| *Nardus stricta* | Poaceae | Graminoid | 7 |
| *Neotinea ustulata* | Orchidaceae | Forb | 4 |
| *Neottia ovata* | Orchidaceae | Forb | 5.8 |
| *Onobrychis viciifolia* | Fabaceae | Forb | 3.2 |
| *Orchis mascula* | Orchidaceae | Forb | 4.2 |
| *Origanum vulgare* | Lamiaceae | Forb | 3.2 |
| *Phleum phleoides* | Poaceae | Graminoid | 2.6 |
| *Phyteuma orbiculare* | Campanulaceae | Forb | 4.7 |
| *Pilosella officinarum* | Compositae | Forb | 3.8 |
| *Pimpinella major* | Apiaceae | Forb | 5.6 |
| *Pimpinella saxifraga* | Apiaceae | Forb | 3.4 |
| *Plantago lanceolata* | Plantaginaceae | Forb | 5 |
| *Plantago media* | Plantaginaceae | Forb | 3.9 |
| *Poa bulbosa* | Poaceae | Graminoid | 2.7 |
| *Poa pratensis* | Poaceae | Graminoid | 5 |
| *Polygala chamaebuxus* | Polygalaceae | Forb | 3 |
| *Potentilla erecta* | Rosaceae | Forb | 7 |
| *Potentilla pusilla* | Rosaceae | Forb | 2 |
| *Potentilla tabernaemontani* | Rosaceae | Forb | 2.7 |
| *Primula veris* | Primulaceae | Forb | 4 |
| *Ranunculus acris* | Ranunculaceae | Forb | 6 |
| *Ranunculus bulbosus* | Ranunculaceae | Forb | 3.2 |
| *Ranunculus polyanthemos* | Ranunculaceae | Forb | 4.2 |
| *Rhinanthus alectorolophus* | Orobanchaceae | Forb | 4 |
| *Rhinanthus minor* | Orobanchaceae | Forb | 5 |
| *Rubus fruticosus* | Rosaceae | Shrub | 6 |
| *Rumex acetosa* | Polygonaceae | Forb | 5 |
| *Rumex acetosella* | Polygonaceae | Forb | 4.4 |
| *Salvia pratensis* | Lamiaceae | Forb | 3.3 |
| *Schedonorus pratensis* | Poaceae | Graminoid | 5.8 |
| *Securigera varia* | Fabaceae | Forb | 4 |
| *Sedum acre* | Crassulaceae | Forb | 1.8 |
| *Sedum album* | Crassulaceae | Forb | 2.2 |
| *Sedum rupestre* | Crassulaceae | Forb | 2 |
| *Sesleria caerulea* | Poaceae | Graminoid | 7.5 |
| *Silene nutans* | Caryophyllaceae | Forb | 3 |
| *Silene vulgaris* | Caryophyllaceae | Forb | 4 |
| *Stachys officinalis* | Lamiaceae | Forb | 4.8 |
| *Stachys recta* | Lamiaceae | Forb | 3 |
| *Stipa pennata* | Poaceae | Graminoid | 2 |
| *Succisa pratensis* | Caprifoliaceae | Forb | 7 |
| *Teucrium chamaedrys* | Lamiaceae | Forb | 2 |
| *Teucrium montanum* | Lamiaceae | Forb | 1 |
| *Thesium alpinum* | Santalaceae | Forb | 4 |
| *Thymus praecox* | Lamiaceae | Shrub | 3 |
| *Thymus pulegioides* | Lamiaceae | Shrub | 4 |
| *Tragopogon pratensis* | Compositae | Forb | 4 |
| *Trifolium arvense* | Fabaceae | Forb | 2.7 |
| *Trifolium campestre* | Fabaceae | Forb | 4 |
| *Trifolium medium* | Fabaceae | Forb | 4 |
| *Trifolium montanum* | Fabaceae | Forb | 3 |
| *Trifolium pratense* | Fabaceae | Forb | 5 |
| *Trifolium repens* | Fabaceae | Forb | 5 |
| *Trisetum flavescens* | Poaceae | Graminoid | 4 |
| *Trollius europaeus* | Ranunculaceae | Forb | 7 |
| *Verbascum lychnitis* | Scrophulariaceae | Forb | 3 |
| *Veronica arvensis* | Plantaginaceae | Forb | 4.3 |
| *Veronica chamaedrys* | Plantaginaceae | Forb | 4.8 |
| *Veronica officinalis* | Plantaginaceae | Forb | 4.2 |
| *Vicia cracca* | Fabaceae | Forb | 5.2 |
| *Vincetoxicum hirundinaria* | Apocynaceae | Forb | 3 |
| *Viola hirta* | Violaceae | Forb | 3.2 |

## References

Bhattacharya, A., & Dunson, D. B. (2011) ‘Sparse Bayesian infinite factor models’, Biometrika, 98, 291-306.

Brooks, S. P., & Gelman, A. (1998). General methods for monitoring convergence of iterative simulations. Journal of computational and graphical statistics, 7, 434-455.

Flegal, J. M., Hughes, J., Vats, D., & Dai, N. (2017). mcmcse: Monte Carlo standard errors for MCMC. Riverside, CA, Denver, CO, Coventry, UK, and Minneapolis, MN. R package version, 1-3.

Gelman, A., & Rubin, D. (1992). Inference from iterative simulation using multiple sequences. Statistical Science, 7, 457-472.

Hengl, T. et al. (2014) ‘SoilGrids1km - Global soil information based on automated mapping’, PLoS ONE, 9, e105992

Karger, D. N. et al. (2017) ‘Climatologies at high resolution for the earth’s land surface areas’, Scientific data, 4, 170122.

Lutz, J. A., Van Wagtendonk, J. W. and Franklin, J. F. (2010) ‘Climatic water deficit, tree species ranges, and climate change in Yosemite National Park’, Journal of Biogeography, 37, 936–950.

Hargreaves, G. H. and Allen, R. G. (2003) ‘History and evaluation of Hargreaves evapotranspiration equation’, Journal of irrigation and Drainage Engineering, 129, 53–63.

Hartig, F. (2019). Package ‘DHARMa’: residual diagnostics for hierarchical (multi-level/mixed) regression models. 2018.

Ovaskainen, O. and Soininen, J. (2011), ‘Making more out of sparse data: hierarchical modeling of species communities’, Ecology, 92, 289-295.

Ovaskainen, O. et al. (2017) ‘How to make more out of community data? A conceptual framework and its implementation as models and software’, Ecology letters, 20, 561–576.

Tikhonov, G. et al. (2017) ‘Using joint species distribution models for evaluating how species-to-species associations depend on the environmental context’, Methods in Ecology and Evolution, 8, 443–452
