# Supplementary material for: Conditional love? Co‐occurrence patterns of drought‐sensitive species in European grasslands are consistent with the stress‐gradient hypothesis
Source: Glob Ecol Biogeogr. 2021 May 31;30(8):1609–20. doi: 10.1111/geb.13323 (PMC8362124; doi:10.1111/geb.13323)
Supplement: Supplementary file 2 — Fig S2 [file GEB-30-1609-s002.pdf]

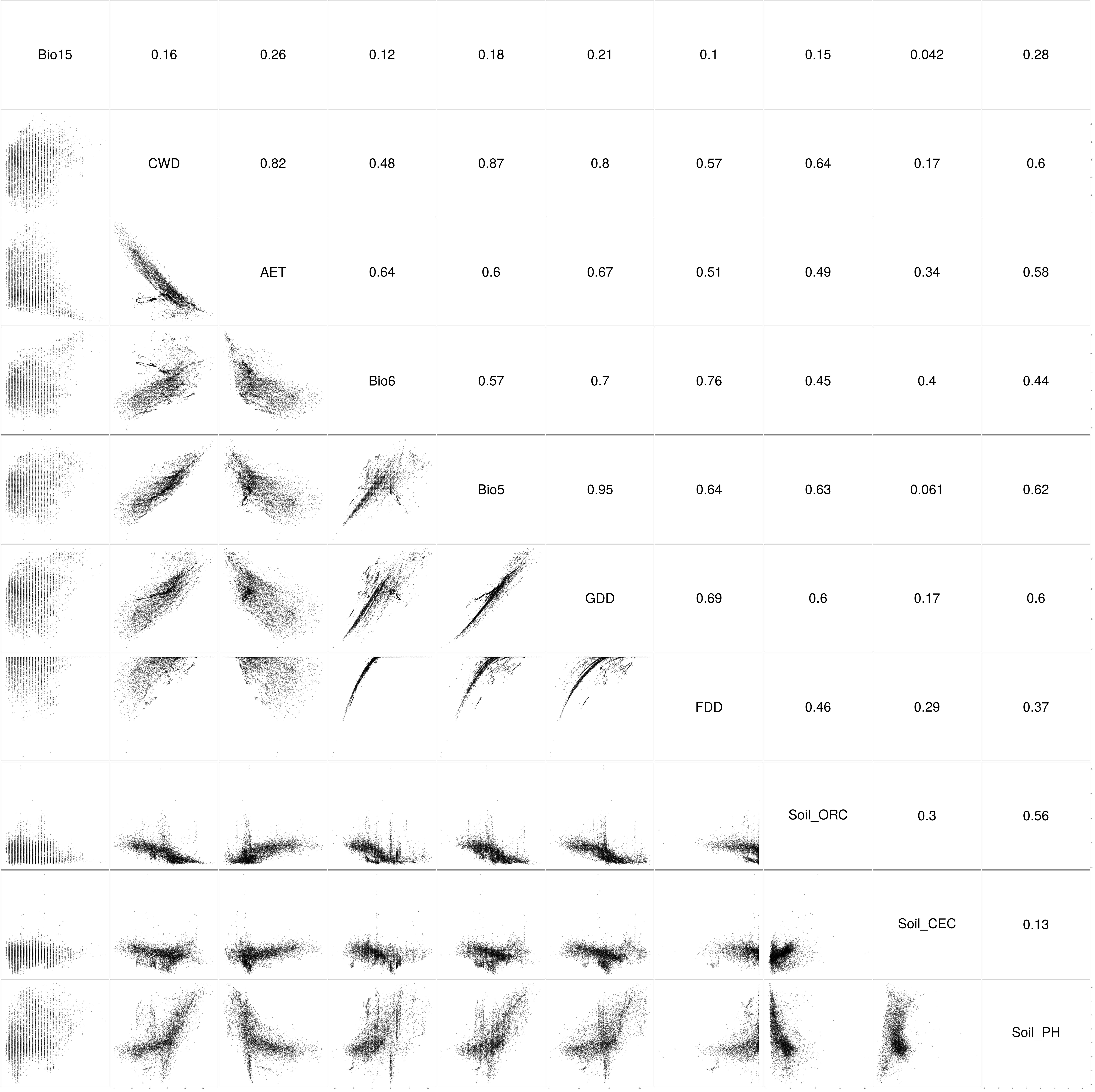
Bio15
0.16
0.26
0.12
0.18
0.21
0.1
0.15
0.042
0.28
CWD
0.82
0.48
0.87
0.8
0.57
0.64
0.17
0.6
AET
0.64
0.6
0.67
0.51
0.49
0.34
0.58
Bio6
0.57
0.7
0.76
0.45
0.4
0.44
Bio5
0.95
0.64
0.63
0.061
0.62
GDD
0.69
0.6
0.17
0.6
FDD
0.46
0.29
0.37
Soil_ORC
0.3
0.56
Soil_CEC
0.13
Soil_PH
